# Effects of Light Condition on Growth and Physiological Characteristics of the Endangered Species *Sedirea japonica* under RCP 6.0 Climate Change Scenarios

**DOI:** 10.3390/plants10091891

**Published:** 2021-09-13

**Authors:** Kyeong Cheol Lee, Jiae An, Jung Eun Hwang, Pyoung Beom Kim, Hyeong Bin Park, Seongjun Kim, Hwan Joon Park, Chang Woo Lee, Byoung-Doo Lee, Nam Young Kim

**Affiliations:** 1 Department of Forestry, Korea National College of Agriculture and Fisheries, Jeonju 54874, Korea; dlrud112@korea.kr; 2 Research Center for Endangered Species, National Institute of Ecology, Yeongyang 36531, Korea; jiae_an@nie.re.kr (J.A.); hwangje@nie.re.kr (J.E.H.); phb1274@nie.re.kr (H.B.P.); dao1229@nie.re.kr (S.K.); rhg9281@nie.re.kr (H.J.P.); jacky903@nie.re.kr (C.W.L.); bdlee@nie.re.kr (B.-D.L.); 3 Wetland Center, National Institute of Ecology, Changnyeong 50303, Korea; normal@nie.re.kr

**Keywords:** climate change, photoinhibition, chlorophyll florescence, CAM plant, CO_2_ exchange rate, SPDS chambers

## Abstract

This study was conducted to evaluate the physiological and growth responses of *Sedirea japonica* cultured in chambers under RCP 6.0 and different light conditions. *S. japonica* was grown in a soil–plant daylight system chamber under two treatments, a control (CO_2_ = 400 ppm) and a climate change treatment (CCT) (CO_2_ = 650 ppm, temperature = control + 3 °C), and three different shading treatments (60%, 90%, and no-shading). *S. japonica* showed the characteristics of typical Crassulacean acid metabolism (CAM) plants. As the shading rate increased, it increased chlorophyll content, leaf area, and leaf dry weight to efficiently absorb and use light. The CCT had a lower CO_2_ absorption rate, stomatal conductance, and growth rate and slightly higher water utilization efficiency than the control. This was because stomatal closure occurred in the CCT to reduce water loss due to a relatively higher temperature. As CO_2_ fixation decreased and consumption increased due to respiration, the overall growth was inhibited. The CCT without shading revealed a dynamic photoinhibition phenomenon showing a significant increase in ABS/RC, TRo/RC, ETo/RC, and DIo/RC and a decrease in PI _ABS_ and DF _ABS_. In this group, leaf, root, and total dry weight, chlorophyll content, and carotenoid content were the worst growth indices.

## 1. Introduction

*Sedirea japonica* is a small epiphytic orchid that grows on a rock or tree and is characterized by 4–10 light greenish-white flowers in a racemose inflorescence between June and August [1]. *S. japonica* is found in Korea, China, Japan, and Taiwan, however, it has almost disappeared in the wild due to indiscriminate harvesting [2,3]. It is registered as an endangered species in Japan and designated as a level 1 endangered species by the Ministry of Environment in South Korea [2,3]. In particular, it is considered to be extinct in the natural condition because no indigenous plant has been reported in the last 20 years, except for the cultivated plants transplanted into nature [3]. The Ministry of Environment has designated it as a priority restoration target species and has initiated a restoration project [3].

Climate change, which is accelerating due to anthropogenic activities, is a major impediment to biodiversity, and it threats the survival of endangered species with high ecological vulnerabilities, such as *S. japonica* [4,5]. The 5th Assessment Report presented by the Intergovernmental Panel on Climate Change (IPCC) indicated that when the global mean temperature increases by 1.5–2.5 °C or more, it will have a negative and irreversible effect on biodiversity and ecosystems, e.g., approximately 20–30% of the animal and plant species included in the assessment would become extinct and their geographical distribution range would be greatly altered [4].

The representative concentration pathways (RCP) scenario, showing a broad range of future climate changes, refers to the degree of influence that changes the energy balance due to radiative forcing, e.g., greenhouse gases [4]. Four RCP scenarios (2.6, 4.5, 6.0, and 8.5) are the most widely used [4]. Among these, RCP 6.0 is a representative intermediate scenario, indicating a condition wherein the greenhouse gas reduction policy has been achieved to some extent. Based on the temperatures recorded between 1986 and 2005, the mean surface temperature of Earth is predicted to increase by 1.4–3.1 °C in 2081 to 2100 [6]. The climate change forecast data based on the Korean Peninsula predicts that carbon dioxide (CO_2_) concentration will increase by 670 ppm and the temperature will increase by 2.7 °C in the RCP 6.0 scenario [7]. For investigations on the effects of climate change conditions on the growth of endangered species, it is necessary to conduct an overall assessment of the vulnerable species so as to preserve them as well as to determine the relationship between physiological characteristics of each species and environmental factors.

An increase in atmospheric CO_2_ concentration is expected to increase the photosynthetic rate and growth due to the CO_2_ fertilization effect [8,9]. Furthermore, the growth of Crassulacean acid metabolism (CAM) plants are also reported to increase [10]. However, the CO_2_ fertilization effect cannot exclude other environmental factors [11], and further investigations on complex environmental changes, including light conditions, need to be conducted.

Until recently, studies on *S. japonica* have conducted lineage and species identification using nucleotide sequences [1], pollination [2], and habitat suitability assessments [3]. However, there are few studies on their physiological and ecological characteristics and the effect of climate change. Therefore, this study aimed to provide the baseline data for establishing conservation strategies for *S. japonica*, an endangered species, by evaluating the effects of climate change (RCP 6.0) and different light conditions on its growth and physiological characteristics.

## 2. Results and Discussion

### 2.1. Comparison of Environmental Conditions

**Figure 1:**
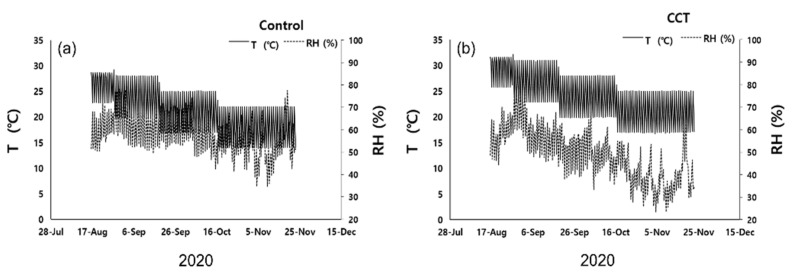
Changes of temperature (T) and relative humidity (RH) of *Sedirea japonica* under control (CO_2_ = 400 ppm) (**a**) and CCT (climate change treatment, CO_2_ = 650 ppm, temperature = control + 3 °C) (**b**) during the experimental period.

The mean daily temperature of the control samples decreased from 25.3 °C in August to 18 °C in November and that of the CCT samples was 2.9 °C higher than that of the control samples on average. Moreover, 60% and 90% shading decreased the room temperature by approximately 1.8 °C and 3.3 °C, respectively, compared to no shading. Although the relative humidity was initially set at 70%, it was not kept constant. During the experiment, the daily mean relative humidity was 58.3% and 49.1% for the control and CCT samples, respectively, showing a 9% difference. In particular, the difference in daily mean relative humidity between the control and CCT samples was not large from August to mid-September; however, it became larger after mid-September (Figure 1).

### 2.2. Daily Change in CO_2_ Absorption Rate and Stomatal Response

**Figure 2:**
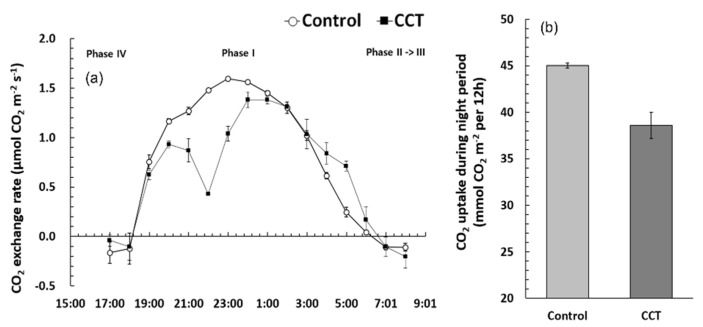
Changes of CO_2_ exchange rate (**a**) and CO_2_ uptake during night period (**b**) of *Sedirea japonica* under control (CO_2_ = 400 ppm) and CCT (climate change treatment, CO_2_ = 650 ppm, temperature = control + 3 °C) conditions. Each value is expressed as the mean ± SD (*n* = 3).

*S. japonica* exhibited the characteristics of a typical CAM plant that absorbs CO_2_ mainly at night (Figure 2). CO_2_ emission and absorption are switched right before sunset (Phase IV). It absorbed a relatively high level of CO_2_ steadily from 7 p.m. (Phase I), and the absorption rate started to rapidly decrease after 5 a.m. (Phase II). It closes the stomata and stops CO_2_ absorption at sunrise (around 6–7 a.m.) and fixes CO_2_ again via the Calvin cycle during the daytime (Phase III) [12]. CO_2_ is fixed mainly from night to before sunrise by phosphoenolpyruvate carboxylase. It is temporarily stored in the vacuoles in the form of malic acid (C4) through oxaloacetic acid, and then it is again fixed to ribulose 1,5-bisphosphate by ribulose-1,5-bisphosphatecarboxylase/oxygenase (RuBisCo) during the daytime [13].

The control samples clearly showed changes in the CO_2_ exchange rate, whereas the CCT samples showed a slightly decreasing absorption rate from 10 to 11 p.m. and then a recovering trend (Figure 2a). In general, the daily carbon assimilation rate of CAM plants is low; approximately 1/2 of that of C3 plants and 1/3 of that of C4 plants [13]; for *S. japonica*, it was up to 1.6 µmol CO_2_ m^−2^s^−1^ (Figure 2).

Various types of CAM plants tend to absorb more CO_2_ as the atmospheric CO_2_ concentration increases [10]. When the atmospheric CO_2_ concentration is doubled, the daily CO_2_ uptake of *Ananas comosus* increased by approximately 15% [14], that of *Agave salmiana* increased by approximately 59% [15], and that of *Phalaenopsis* increased by approximately 25 to 31% [16]. This reaction is highly related to the activity of RuBisCo in Phases II and IV, where the stomata open and close. High atmospheric CO_2_ concentration increases the activity of RuBisCo, thus increasing the amount of CO_2_ fixation [10,17].

Upon comparing the CO_2_ uptake by *S. japonica* during the night (Figure 2b), it was found that the control samples fixed 45.0 mmol CO_2_·m^−2^ and the CCT samples fixed 38.6 mmol CO_2_·m^−2^, indicating that the control samples absorbed 17% more CO_2_. Previous studies on the response of CAM plants to the increase in atmospheric CO_2_ concentration were conducted under the same temperature conditions, however, the CCT showed increases in temperature of ≥3 °C compared to the control and increased atmospheric CO_2_ concentration in the chamber. In other words, it can be considered that, when *S. japonica* samples were grown under a relatively high temperature for a long time, the net daily CO_2_ uptake decreased because more stomata closed to prevent loss of moisture. In some CAM plants, such as *Clusia uvitana* and *Portulacaria afra*, it has been reported that CO_2_ uptake at night becomes stagnant or decreases under a moisture stress condition, even if the atmospheric CO_2_ concentration increases [18,19]. These results also imply that CO_2_ uptake is closely related to stomatal opening and closing.

**Figure 3:**
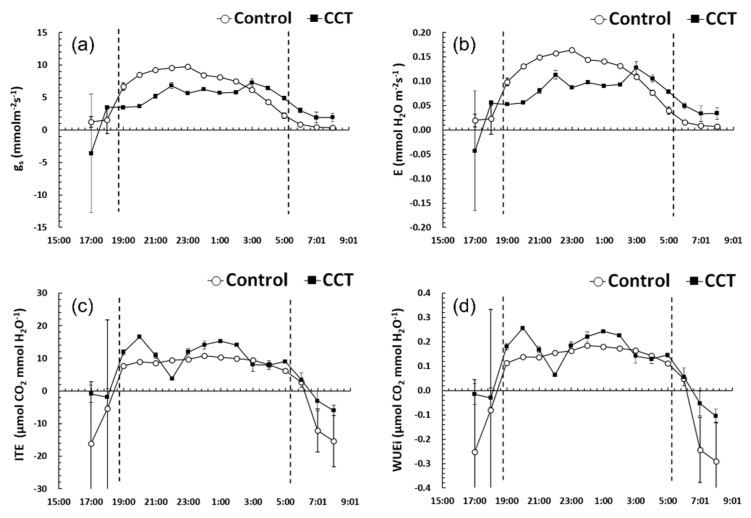
Changes of stomatal conductance (g_s_) (**a**), stomatal transpiration rate (E) (**b**), instantaneous transpiration efficiency (ITE) (**c**), and intrinsic water use efficiency (WUEi) (**d**) of *Sedirea japonica* under control (CO_2_ = 400 ppm) and CCT (climate change treatment, CO_2_ = 650 ppm, temperature = control + 3 °C) conditions. Each value is expressed as the mean ± SD (*n* = 3).

In the control samples, g_s_ and E rapidly increased after 6 p.m., maintained a high value, and then considerably decreased after 3 a.m. In contrast, in the CCT samples, the increase was relatively small, and the values were generally lower than those of the control samples until 3 a.m. (Figure 3a,b). This trend showed that the CCT samples could not absorb CO_2_ in the atmosphere smoothly because they were not able to quickly switch from closed stomata during the daytime to opened stomata during the night compared to the controls. This is believed to be the result of adaptation to stomatal opening and closing to prevent loss of moisture while growing for a long time under higher mean temperature conditions (higher than at least 3 °C) than the control. The WUEi and ITE of the CCT samples were higher than those of the control, supporting this result (Figure 3c,d). A similar trend was also observed for other CAM species such as *Agave deserti* [20], *Opuntia ficus-indica* [21], and *Phalaenopsis* spp. [22].

### 2.3. Chlorophyll Fluorescence Response

The chlorophyll fluorescence analysis determined the imbalance between the light energy absorbed by photochemical reactions and the energy used in electron transfer reactions. In particular, the OKJIP curve shows physiological responses to various environmental stresses quantitatively through changes in energy flow in photosystem II [13,23].

**Figure 4:**
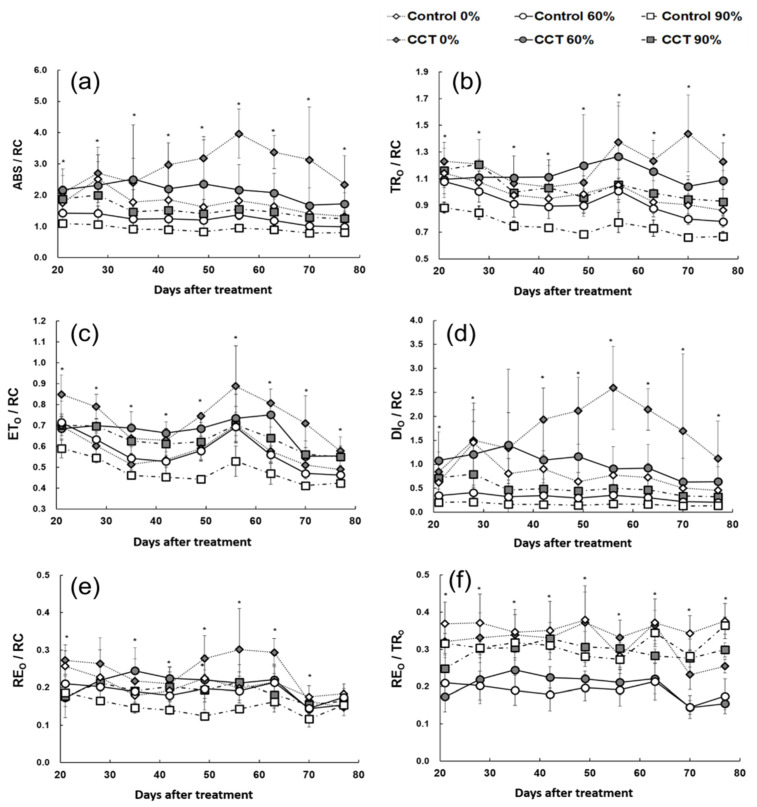
Effects of shading level on several chlorophyll fluorescence parameters (ABS/RC (**a**), TR_0_/RC (**b**), ET_0_/RC (**c**), DI_0_/RC (**d**), RE_0_/RC (**e**), and RE_0_/TR_0_ (**f**)) of *Sedirea japonica* under control (CO_2_ = 400 ppm) and CCT (climate change treatment, CO_2_ = 650 ppm, temperature = control + 3 °C) conditions. Each value is expressed as the mean ± SD (*n* = 5). The asterisk indicates significance at *p* < 0.05.

When monitoring the chlorophyll fluorescence response according to the climate change conditions and shading rate, ABS/RC, TRo/RC, ETo/RC, and DIo/RC, which indicate the change in energy flow per reaction center, were the lowest in the 90% shading control samples (control 90%). In contrast, the ABS/RC and DIo/RC of the CCT samples without shading (CCT 0%) were significantly (*p* < 0.05) higher than other treatments from day 42, and ETo/RC and TRo/RC were also the highest around day 55 after treatment (Figure 4a–d).

ABS/RC indicates light energy absorbed per reaction center, and DIo/RC refers to the light energy dissipated as heat [23]. The increase in ABS/RC and DIo/RC in the CCT 0% treatment samples can be understood as a mechanism to prevent damage to the photosynthetic apparatus under strong light and relatively high temperature conditions [24,25]. It can be seen that it deactivates the reaction center and dissipates the excitation energy as heat [24,25].

Moreover, TRo/RC and ETo/RC refer to changes in light energy captured in photosystem II per reaction center and changes in energy transferred to the electron transport system afterward [23]. It was found that although *S. japonica* has a primary protection mechanism against strong light, it could not block the excessive inflow of light energy under the CCT 0% treatment for a long time, and it affected the overall photoreaction process. *Phalaenopsis* “Edessa”, a CAM plant belonging to *Orchidaceae*, showed a similar overall energy flow pattern under drought stress [25]. The decrease in ABS/RC, TRo/RC, ETo/RC, and DIo/RC observed around day 79 after treatment was presumed to be a process of gradually recovering the decrease in efficiency due to stress as the sun’s radiation decreased over time (Figure 4a–d).

REo/RC shows the electron transport energy flow to photosystem I [23], showing a similar trend to TRo/RC and ETo/RC (Figure 4e). Additionally, the overall photosynthetic activity was low in the control 90% treatment samples due to excessively low light condition without the appropriate temperature condition required for metabolic activity. It can be recovered when sufficient light energy is supplied again. From the relatively high REo/TRo, it is expected that the reaction of transferring the light energy captured in photosystem II to photosystem I was relatively efficient (Figure 4f).

**Figure 5:**
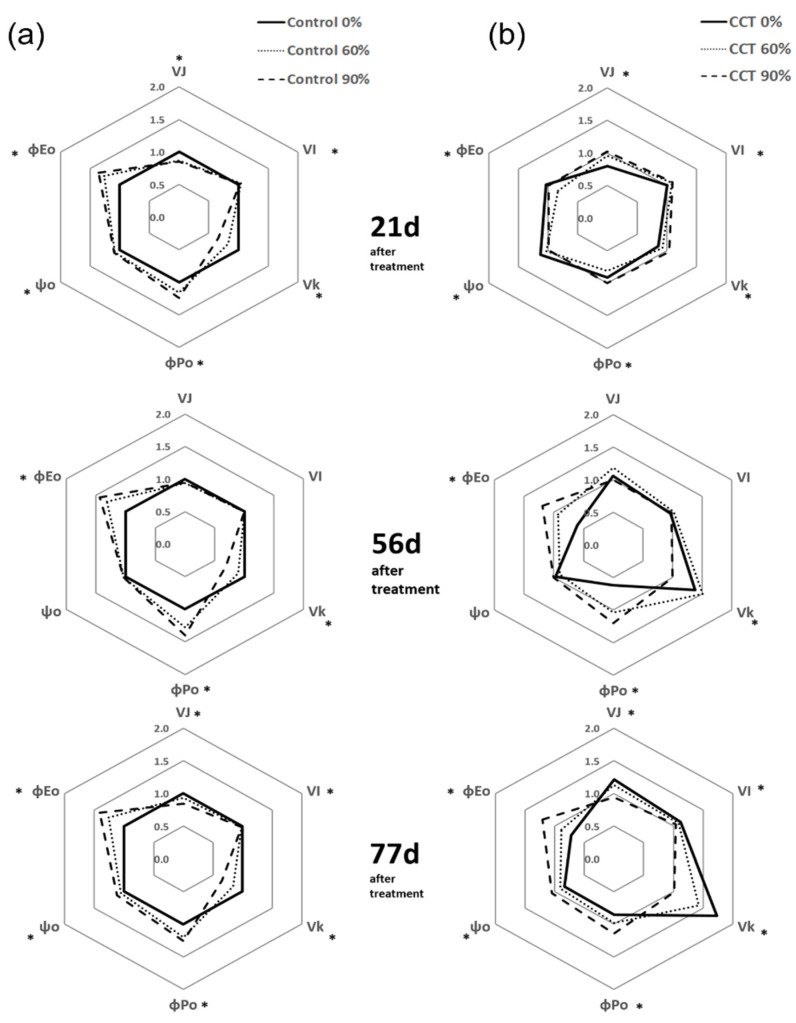
Radar plots of several fluorescence parameters (VJ, VI, Vk, ΦPO, ΦEO, and ΨO) of *Sedirea japonica* under control (CO_2_ = 400 ppm) (**a**) and CCT (climate change treatment, CO_2_ = 650 ppm, temperature = control + 3 °C) (**b**) conditions 21, 56, and 77 days after the treatment. The asterisk indicates significance at *p* < 0.05.

In the case of the control samples, Ψ_O_ did not considerably change on days 21, 56, and 77 after treatment, whereas Φ_PO_ and Φ_EO_ slightly increased as the shading rate increased (Figure 5a). Φ_PO_ indicates the maximum quantum yield in the initial photochemical reaction, and Φ_EO_ shows the quantum yield of electron transport after Q_A_^-^, the primary electron acceptor in photosystem II [23]. Thus, it was possible to know that the electron transport efficiency increased due to the shading treatment. However, some indices of the CCT samples showed relatively large differences around day 56 after treatment. In the CCT 0% treatment, Φ_PO_ decreased by 38% and Φ_EO_ decreased by 40% compared to the control 0% treatment. In contrast, V_k_ increased by 38% (Figure 5). Although the other indices of the CCT 60% treatment did not show a large difference, V_k_ increased by approximately 50%. The increase in V_k_ indicated that electron transfer was inhibited by the deactivation of the oxygen-evolving complex after the Q_A_^-^ in photosystem II [24]. After 77 days, the control samples showed a similar tendency to that observed around day 56 according to the shading rate, whereas the decrease in Φ_PO_ and Φ_EO_ and the increase in V_k_ in the CCT 0% treatment were much greater than those observed on day 56 (Figure 5). These results revealed that the overall electron transfer efficiency was inhibited due to the decrease in Φ_PO_ and Φ_EO_ and the increase in V_k_ in the CCT compared to the control samples, and the shading treatment slightly improved this tendency.

**Figure 6:**
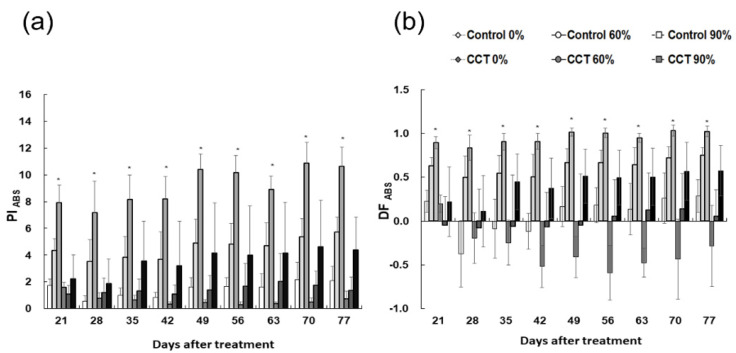
Effects of shading level on PI _ABS_ (**a**) and DF _ABS_ (**b**) of *Sedirea japonica* under control (CO_2_ = 400 ppm) and CCT (climate change treatment, CO_2_ = 650 ppm, temperature = control + 3 °C) conditions. Vertical bars are mean ± SD (*n* = 5). The asterisk indicates significance at *p* < 0.05.

The activity of photosystem II of *S. japonica* was observed according to climate change conditions using chlorophyll fluorescence analysis (Figure 6). PI_ABS_ and DF_ABS_, the vitality indices of the photosynthetic apparatus, greatly increased with a higher shading rate in both the control and CCT samples [23]. PI_ABS_ refers to the energy conservation efficiency in the process of reducing electron carriers by using absorbed light energy, and DF_ABS_ indicates the momentum in the electron transport process [25,26,27]. PI_ABS_ and DF_ABS_ in *S. japonica* around day 35 after treatment tended to be control 90% > control 60% > CCT 90% > control 0% ≧ CCT 60% > CCT 0%. In particular, a distinct decrease was observed in CCT 0% treatment (Figure 6). The result showing a drastic decrease in the overall activity of photosystem II, such as energy conservation efficiency and momentum, in the electron transfer process can be understood as a photoinhibition that occurs when growing in a strong light environment for a long time. It was distinct when the atmospheric CO_2_ concentration and temperature were relatively high.

Additionally, it is believed that when it grows under insufficient light condition due to shading treatment, an adaptive reaction appears to process light energy more efficiently by increasing the activity of photosystem II, such as PI_ABS_ and DF_ABS_. The control samples showed a higher tendency than the CCT, indicating a relatively sensitive response. *Phalaenopsis* “Edessa” also showed that plants growing in low light condition had higher PI_ABS_ [25].

### 2.4. Chlorophyll and Carotenoid Contents

The chlorophyll content of *S. japonica* tended to increase as the shading rate increased (*p* < 0.05). The CCT samples tended to show lower values than the control under the same shading conditions (Table 1). However, the interaction between climate change conditions and shading treatment was not significant (*p* > 0.05).

In general, as an adaptation to absorb more light under low light condition, the increase in chlorophyll b constituting the light-harvesting chl–protein complex is generally larger than the increase in chlorophyll a, which is mainly bound to the reaction center; therefore, chlorophyll a/b tends to decrease [28,29]; *S. japonica* generally showed the same tendency, except for control 60% treatment (Table 1). In other words, it can be understood as an adaptation process of increasing the antenna pigment to increase the light-receiving rate and the efficiency of using light while growing in a relatively insufficient light environment [30,31]. For 0% and 90% shading treatments, the chlorophyll a/b of the CCT samples tended to be lower than that of the controls. This was due to the difference in the decrease in chlorophyll a, and it could decrease the activity of the carbon fixation system [32].

Carotenoids perform a photoprotective function that quickly eliminates the excited state of chlorophyll under stress conditions such as strong light environments [33]. However, carotenoid content increased with a higher shading rate for *S. japonica* (Table 1). This is believed to be because carotenoids acted more as an auxiliary pigment rather than focusing on the photoprotective function.

### 2.5. Growth Characteristics

The number and thickness of *S. japonica* leaves were not significantly different (*p* > 0.05). Leaf dry weight, leaf area, SLA, and LAR were significantly different among the shading treatments (*p* < 0.05). The increase in leaf dry weight, leaf area, and shading rate could be understood as a general reaction to increasing the efficiency of using light (Table 2). Moreover, root dry weight, total dry weight, T/R ratio, SLA, LAR, and LWR were significantly different among climate change conditions (*p* < 0.05). However, the interaction between climate change conditions and shading was not significant (*p* > 0.05).

For the leaf thickness of the control samples, the 0% shading treatment tended to be lower than that of the 60% and 90% shading treatments, which is considered to be because the leaf growth of the 0% shading treatment was relatively lower compared to the 60% and 90% shading treatments, so no significant features were revealed.

For many CAM plants, the development of vacuoles, storing malate in a high CO_2_ concentration environment, can affect CO_2_ uptake [34]. Cui et al. [21] reported that the overall plant size and thickness increased as they became more succulent. This trend was not observed in *S. japonica* (Table 2). *Phalaenopsis* (*Doritaenopsis* Queen Beer ‘Mantefon’), a plant similar to *S. japonica*, also did not show the development of leaf thickness [22].

The dry weight of *S. japonica* was lower in the CCT than in the control samples. In particular, root dry weight showed a large difference to have a great influence on the T/R ratio and the total dry weight (Table 2). These results were different from the generally known trends. Drennan and Nobel [10] reported that 10 CAM plants, including *A. deserti*, had higher biomass and CO_2_ uptake in a high CO_2_ environment (more than double of the control). It is known that C3 and C4 plants often show carbon fertilization effects under high atmospheric CO_2_ conditions [8,9,35]. However, the biomass of some plants, including *A. comosus* [36], slightly decreased in high CO_2_ concentration environments, indicating that there are differences in adaptation between species. Kim et al. [37] reported that *Phalaenopsis* “Fuller’s Pink Swallow” did not show a significant difference in shoot biomass and dry weight when CO_2_ concentration was increased from 450 to 800 ppm; root dry weight was also not different (1.92 g and 1.96 g, respectively).

The decrease in the overall growth of *S. japonica* even under increased atmospheric CO_2_ concentration could be highly related to the difference in CO_2_ uptake (Table 2). It is believed that material consumption increased due to respiration because atmospheric temperature (≥3 °C), as well as atmospheric CO_2_ concentration, was increased in this study, unlike previous studies, which mainly increased the atmospheric CO_2_ concentration. In other words, it can be considered that stomatal closure to reduce loss of moisture in response to relatively high temperature reduced CO_2_ fixation and increased consumption due to respiration, leading to a decrease in overall growth. Increased atmospheric CO_2_ concentration and temperature-induced water stress to *Populus alba × glandulosa*, a C3 plant, and a decrease in the activities of the photochemical and carbon fixation systems due to this resulted in a decrease in photosynthetic capacity and growth rate [32]. The growth of yellow rose mallow, an endangered species just like *S. japonica*, would be limited by the amount of light in higher atmospheric CO_2_ concentration and temperature conditions [5].

These results indicated that increased atmospheric CO_2_ concentration and temperature should be considered to evaluate the ecological adaptation of plants to climate change and global warming. It can be assumed that a response to reduce water loss and an increase in respiration can offset the benefit of carbon fertilization.

## 3. Materials and Methods

### 3.1. Materials and Experimental Design

In this study, 100 *S. japonica* samples proliferated via tissue culture at the Research Center for Endangered Species of the National Institute of Ecology in 2020 were used. Each *S. japonica* sample was placed at the center of a 10-cm diameter pot filled with moss. The samples were carefully grown and bottom irrigation was used to prevent drying of the moss.

The study was conducted in soil–plant daylight system (SPDS) chambers at the Climate Change Education Center of the Korea National College of Agriculture and Fisheries to provide atmospheric CO_2_ concentration and temperature conditions according to the climate change scenario. The experiment was conducted from 24 August to 13 November 2020, while performing periodic observations.

The samples were divided into control and climate change treatments (CCT) according to the RCP 6.0 scenario. The control samples were maintained under CO_2_ concentration of 400 ppm, and the temperature inside the chamber was periodically modified according to the meteorological conditions in Jeonju in the past 3 years. For the CCT samples, CO_2_ concentration was maintained at 650 ppm, and the temperature inside the chamber was maintained as that used for the control + 3 °C. Moreover, a shade was installed to provide no shading, 60% shading, and 90% shading treatments in both the control and CCT chambers, and the light was measured intensity using a photo-radiometer (HD 2102.1, Delta OHM, Italy) on midday of 20 August, which showed 2062 ± 125 µmol m^−2^s^−1^ in the control, 863 ± 74 µmol m^−2^s^−1^ in the 60% shading, and 179 ± 52 µmol m^−2^s^−1^ in the 90% shading (*n* = 10). Then, 10 samples were placed for each treatment to examine the growth and physiological response of *S. japonica* under different light conditions in the climate change scenario. The changes in temperature and relative humidity were monitored using the meteorological observation sensor installed in the chamber, and the internal environment due to the shading treatment was investigated thrice at around 12 p.m. on 15 September using a portable photometer and temperature and humidity meter.

### 3.2. Daily Changes in CO_2_ Absorption Rate and Stomatal Response

The CO_2_ absorption rate of *S. japonica* by climate change condition was investigated for the control samples and those grown under non-shading CCT conditions. The CO_2_ exchange rate and stomatal conductance (g_s_) were measured using a portable photosynthesis system (Li-6800, Li-Cor Inc., Lincoln, NE, USA), with three replicate plants in each treatment from 4 p.m. to 8 a.m. from 13 November to 30 November. During the measurements, ambient light was provided using the clear-top leaf chamber (6800-12A, Li-Cor Inc., Lincoln, NE, USA), and photosynthetic photon flux density has been measured concurrently, showing the values of 140~250 µmol m^−2^s^−1^ (4 p.m. to 7 p.m.) and 0~8 µmol m^−2^s^−1^ (7 p.m. to 6 a.m.). Moreover, CO_2_ uptake, intrinsic water use efficiency (WUE_i_), and instantaneous transpiration efficiency (ITE) at night (7 p.m. to 6 a.m.; 12 h) were calculated from the measurement [22,38,39]. For the measurement, relative humidity was 60%, the amount of air inflow into the chamber was 600 μmols^−1^, and CO_2_ concentration was 400 and 650 ppm based on the mean concentration of each treatment. The leaf temperature was set at 25 °C for the control and 28 °C for the treatment samples.

### 3.3. Chlorophyll Fluorescence Response

Chlorophyll fluorescence response was evaluated using polyphasic increase in chlorophyll a fluorescence transients (OKJIP) analysis at 1-week intervals from day 21 after initiating the shading treatment and CCT. Dark-adapted leaves were irradiated with 3500 µmol m^−2^s^−1^ light for 20 min using a Plant Efficiency Analyzer (Hansatech Instrument Ltd., King’s Lynn, England), and chlorophyll fluorescence densities were measured at 50 µs (O stage), 300 µs (K stage), 2 ms (J stage), 30 ms (I stage), and 500 ms (P stage). All leaf measurements were done using the uppermost mature leaf of each of the five replicate plants. Biophysical parameters (V_J_, V_I_, V_k_, Φ_PO_, Φ_EO_, Ψ_O_, ABS/RC, DIo/RC, TRo/RC, ETo/RC, RE_0_/RC, RE_0_/TR_0_, PI_ABS_, and DF_ABS_; Table 3) were calculated from the results of OKJIP analysis [23,24].

### 3.4. Chlorophyll and Carotenoid Contents

After completing all physiological response tests, three leaves were randomly collected from each treatment, and 0.1 g of the leaf blade was placed in a 20 mL glass bottle containing 10 mL of dimethyl sulfoxide solution. The pigments were extracted for 6 h in a thermostat set at 60 °C [40]. The absorbance of the solution was measured at wavelengths of 663, 645, and 470 nm using a UV/VIS spectrophotometer (HP 8453, Hewlett-Packard, New York, NY, USA) to estimate chlorophyll a, b, a + b, and carotenoid contents [41,42].

### 3.5. Growth Characteristics

After evaluating all the physiological responses, the number of leaves, leaf thickness, leaf area, leaf dry weight, and root dry weight were measured. Leaf thickness was measured at the utmost center of the leaf using Vernier calipers, and the dry weight was measured after drying the leaf at 80 °C for 48 h in a dryer (DS-80-5, Dasol Scientific Co. Ltd., Gyeonggido, Korea). Thereafter, tree/root ratio (T/R ratio), specific leaf area (SLA), leaf area ratio (LAR), and leaf weight ratio (LWR) were calculated. One-way analysis of variance (ANOVA) was performed for the CO_2_ exchange rate and chlorophyll fluorescence response by leaf temperature, and the significance among each treatment was determined at 5% (Duncan’s multiple range test). The relation among chlorophyll content, carotenoid content, and growth response effects due to climate change and photoenvironment was assessed using two-way ANOVA. SPSS Statistics Program (Version 19.0) was used for all statistical analyses.

## 4. Conclusions

*S. japonica* showed characteristics that are typical of CAM plants that fix CO_2_ at night. As the shading rate increased, the chlorophyll a, b, a + b, and carotenoid content, the leaf area, and the leaf dry weight increased to absorb and use light efficiently. Moreover, the CCT samples showed a lower CO_2_ absorption rate, CO_2_ uptake, stomatal conductance, and biomass growth and a higher water utilization efficiency than the control. This could be because of stomatal closure in the CCT to reduce water loss due to a relatively higher temperature condition, which reduced CO_2_ fixation and increased consumption due to respiration, which could inhibit the overall growth. In particular, the CCT 0% treatment revealed a dynamic photoinhibition phenomenon showing a significant increase in ABS/RC, TRo/RC, ETo/RC, and DIo/RC and a decrease in PI_ABS_ and DF_ABS_. Furthermore, the leaf dry weight, root dry weight, total dry weight, chlorophyll content, and carotenoid content were the worst growth indices. In conclusion, the RCP 6.0 condition that increases atmospheric CO_2_ concentration and temperature at the same time requires 60% or 90% shading treatment, and no shading provides the least favorable growth condition.

## Figures and Tables

**Table 1:** Effects of shading level on chlorophyll and carotenoid contents of *Sedirea japonica* under control (CO_2_ = 400 ppm) and CCT (climate change treatment, CO_2_ = 650 ppm, temperature = control + 3 °C) conditions.

Treatment	Chlorophyll Content (mg g^−1^)			Carotenoid Content (mg g^−1^)	Chl a/b	T chl/Car
	a	b	a + b			
Control 0%	2.95 ± 0.36	0.60 ± 0.13	3.55 ± 0.49	1.17 ± 0.12	4.99 ± 0.58	3.02 ± 0.15
Control 60%	4.12 ± 0.81	1.26 ± 0.17	5.37 ± 0.66	1.40 ± 0.20	3.38 ± 1.06	3.86 ± 0.07
Control 90%	6.10 ± 0.52	1.35 ± 0.22	7.44 ± 0.64	1.66 ± 0.11	4.60 ± 0.82	4.48 ± 0.32
CCT 0%	2.86 ± 0.40	0.60 ± 0.04	3.46 ± 0.40	1.09 ± 0.19	4.81 ± 0.76	3.19 ± 0.22
CCT 60%	4.26 ± 0.46	1.00 ± 0.18	5.26 ± 0.59	1.25 ± 0.14	4.29 ± 0.50	4.20 ± 0.26
CCT 90%	5.62 ± 0.07	1.41 ± 0.03	7.03 ± 0.10	1.56 ± 0.04	3.99 ± 0.09	4.53 ± 0.05
CCT	ns	ns	ns	*	ns	*
Shading	**	**	**	**	*	**
CCT × Shading	ns	ns	ns	ns	ns	ns

Each value is expressed as the mean ± SD (*n* = 5). * *p* ≤ 0.05, ** *p* ≤ 0.001, and ns: non-significance. CCT: climate change treatment, Chl a: chlorophyll a, Chl b: chlorophyll b, and T chl: total chlorophyll content.

**Table 2:** Effects of shading level on growth characteristics of *Sedirea japonica* under control (CO_2_ = 400 ppm) and CCT (climate change treatment, CO_2_ = 650 ppm, temperature = control + 3 °C) conditions.

Treatment	No. of Leaves	Dry Mass Production (g)			Leaf Thickness (mm)	Leaf Area (cm^2^)	T/R Ratio (g g^−1^)	SLA (cm^2^ g^−1^)	LAR (g g^−1^)	LWR (g g^−1^)
		**Leaf**	**Root**	**Total**						
Control 0%	4.6 ± 1.3	0.22 ± 0.09	0.32 ± 0.1	0.54 ± 0.19	1.50 ± 0.11	39.6 ± 15.8	0.66 ± 0.14	187.5 ± 29.6	73.0 ± 6.6	0.39 ± 0.05
Control 60%	5.0 ± 1.0	0.31 ± 0.05	0.40 ± 0.09	0.72 ± 0.13	1.65 ± 0.08	59.8 ± 6.8	0.80 ± 0.12	191.1 ± 13	85.0 ± 12.1	0.44 ± 0.04
Control 90%	5.6 ± 1.1	0.33 ± 0.06	0.39 ± 0.03	0.72 ± 0.08	1.63 ± 0.12	68.9 ± 8.1	0.87 ± 0.12	207.6 ± 15.5	95.9 ± 7.5	0.46 ± 0.03
CCT 0%	4.6 ± 0.6	0.22 ± 0.09	0.24 ± 0.10	0.46 ± 0.19	1.64 ± 0.11	45.3 ± 12.9	0.96 ± 0.14	211.1 ± 24.7	103.2 ± 16.6	0.49 ± 0.03
CCT 60%	4.4 ± 0.9	0.23 ± 0.05	0.23 ± 0.04	0.46 ± 0.09	1.61 ± 0.11	53.5 ± 6.7	0.98 ± 0.06	239.5 ± 28.7	118.0 ± 11.5	0.50 ± 0.02
CCT 90%	4.4 ± 0.9	0.27 ± 0.08	0.27 ± 0.06	0.53 ± 0.14	1.58 ± 0.05	64.0 ± 15.9	0.99 ± 0.08	242 ± 13.9	120.2 ± 4.3	0.50 ± 0.02
CCT	ns	ns	**	*	ns	ns	**	**	**	**
Shading	ns	*	ns	ns	ns	**	ns	*	*	ns
CCT × Shading	ns	ns	ns	ns	ns	ns	ns	ns	ns	ns

Each value is expressed as the mean ± SD (*n* = 5). * *p* ≤ 0.05, ** *p* ≤ 0.001, and ns: non-significance.

**Table 3:** Summary of chlorophyll fluorescence parameters from OKJIP test.

Parameters	Description
V_J_	Relative variable fluorescence at the J-step
V_I_	Relative variable fluorescence at the I-step
V_k_	Relative variable fluorescence at the k-step
Φ_PO_ (= TRo/ABS)	Probability that an absorbed photon leads to reduction further than Q_A_^-^
Φ_EO_ (= ETo/ABS)	Probability that an absorbed photon leads to electron transport further than Q_A_^-^
Ψ_O_ (= ETo/TRo)	Probability that an absorbed photon leads to reduction of Q_A_^-^
ABS/RC	Absorption flux per reaction center
TR_0_/RC	Trapped energy flux per reaction center
ET_0_/RC	Electron transport flux from QA to QB per reaction center
DI_0_/RC	Energy dissipation flux per reaction center
RE_0_/RC	Electron transport flux until PSI acceptors per reaction center
RE_0_/TR_0_	Efficiency with which a trapped exciton can move an electron into the electron transport chain from QA–to the PSI end electron acceptors
PI_ABS_	Performance index on absorption basis.
DF_ABS_	Driving force on absorption basis.

## Data Availability

Data is contained within the article.
